# Decision Making in Concurrent Multitasking: Do People Adapt to Task Interference?

**DOI:** 10.1371/journal.pone.0079583

**Published:** 2013-11-11

**Authors:** Menno Nijboer, Niels A. Taatgen, Annelies Brands, Jelmer P. Borst, Hedderik van Rijn

**Affiliations:** 1 Department of Artificial Intelligence, University of Groningen, Groningen, The Netherlands; 2 Department of Psychology, Carnegie Mellon University, Pittsburgh, Pennsylvania, United States of America; 3 Department of Experimental Psychology, University of Groningen, Groningen, The Netherlands; University of Tokyo, Japan

## Abstract

While multitasking has received a great deal of attention from researchers, we still know little about how well people adapt their behavior to multitasking demands. In three experiments, participants were presented with a multicolumn subtraction task, which required working memory in half of the trials. This primary task had to be combined with a secondary task requiring either working memory or visual attention, resulting in different types of interference. Before each trial, participants were asked to choose which secondary task they wanted to perform concurrently with the primary task. We predicted that if people seek to maximize performance or minimize effort required to perform the dual task, they choose task combinations that minimize interference. While performance data showed that the predicted optimal task combinations indeed resulted in minimal interference between tasks, the preferential choice data showed that a third of participants did not show any adaptation, and for the remainder it took a considerable number of trials before the optimal task combinations were chosen consistently. On the basis of these results we argue that, while in principle people are able to adapt their behavior according to multitasking demands, selection of the most efficient combination of strategies is not an automatic process.

## Introduction

Multitasking has become a staple of modern society: its influence reaches into just about every aspect of our daily lives. The prevalence of multitasking affects the grades of students [1], the way jobs are performed [2], our safety during driving ([3]–[5]; for an overview see [6]), and even performance during sports [7].

Multitasking research has focused on determining the performance cost of executing several tasks concurrently compared to performing a single task. One important part of multitasking behavior that has received less attention is how people determine which activities to do simultaneously, and at what time. Factors that are important in these decisions are intrinsic motivation (enjoyment, expertise), and personality traits such as impulsivity or sensation seeking [8]. Some of these factors could influence the utility [9] of a task, which determines how likely a task is chosen in a multitasking context. This raises an interesting question: Is utility purely based on aspects of the task itself, and therefore independent of multitasking, or can the multitasking context change the utility of tasks and therefore the decision process?

We hypothesize that task utility can also be influenced by how effectively a second task combines with the primary activity: some combinations of tasks may decrease overall performance, while other tasks do not. If people want to maximize their utility, they should combine tasks that minimize the performance decrement. Although there is some evidence for this from the area of sequential multitasking (i.e., people alternating between tasks [10], [11]), almost no data is available in the context of concurrent multitasking: research has been limited to the influence of task priority imposed by instructions to the participants [12].

In this paper we investigate whether people adapt their choices in concurrent multitasking to combinations of tasks that work together well, even though it is not immediately obvious what these combinations are. Furthermore, we examine whether this adaptation is part of a learning process, or determined instantly.

### Interference in Multitasking

The decrement in performance that can occur when multiple tasks are performed concurrently is typically attributed to interference between the tasks. Theories regarding the effect of interference can roughly be divided into theories emphasizing processing bottlenecks versus theories emphasizing capacity sharing. According to bottleneck theories, certain processing stages (e.g., perception, response) cannot be performed in parallel: when two tasks require such a stage at the same time, one task will be delayed until the other task no longer requires it [13]. Proposed bottleneck theories hold different views regarding which stages or resources can become a bottleneck, and which can be used in parallel [13]–[16].

Capacity sharing theories state that resources can be shared by tasks. However, when two tasks have to share the capacity of a single resource, performance degrades [17]. A well known account of this type is multiple resource theory [18]. According to Wickens, interference increases when tasks share more resources. These resources can be cognitive or response-related stages, but also sensory modalities or information channels. While this gives us a measure of the amount of interference we can expect, it does not explain the mechanism that leads to the performance decrease.

The bottleneck and capacity sharing theories have both influenced the development of cognitive architectures. This resulted in several different computational accounts of multitasking interference. In EPIC (Executive-Process Interactive Control) [19], all central cognitive resources (e.g., declarative memory, production memory) can, but peripheral resources (e.g., vision, motor) can not be used by multiple tasks at the same time. Use of the resources is accomplished through a decision rule system, and serial behavior is a result of a combination of peripheral bottlenecks and a strategy that interleaves production rules of multiple tasks.

A more recent explanation of multitasking interference is threaded cognition [20]. According to threaded cognition, all cognitive resources (i.e., visual perception, motor control, working memory) can only be used by a single process at any given time. Interference will occur when use of a resource by one process will delay another. Task scheduling is achieved by a straight-forward interleaving process: whenever a task needs a particular resource and that resource is not in use by another task, it can use it, otherwise it has to wait. Threaded cognition has been used to explain a variety of multitasking results [14], [21], [22], and is in line with recent investigations into multitasking interference, which identify both serial and parallel components in task processing [23]. This led us to use predictions from threaded cognition to develop a paradigm suitable for investigating how people adapt their multitasking behavior.

### Paradigm

To test whether people adapt their choices to minimize multitasking interference, we developed a paradigm where the expected severity of multitasking interference was varied between four possible task combinations. Participants were given a fixed primary task, multicolumn subtraction, which had to be performed in every trial. The subtraction task consisted of ten columns that had to be solved digit-by-digit in standard right-to-left order. There were two types of subtraction problems: in the easy condition all upper-term digits were larger than the corresponding lower-term digits. Participants therefore did not need to remember any carries in order to solve the problem. In hard condition, six of the ten columns required the participant to perform a carry operation. The subtraction task requires visual (attention and processing), manual (motor control of the hands), as well as declarative memory (retrieving facts about subtracting numbers) resources.

The difference between the two subtraction conditions is that in the hard condition the state of the carry must be maintained between the columns. Based on earlier research we believe this carry to be stored in working memory (WM) [14]. The concept of WM we employ is very similar to that of the focus of attention [14], [24], and is based on the memory system present in ACT-R [25]. This focal WM can contain only a single chunk of information. When multiple chunks have to be remembered, only one chunk will be in the focus, and the rest will be in declarative memory (DM). Thus, requiring WM-like access to multiple chunks will require that chunks be swapped between focal WM and DM. As swapping from DM takes time (in the order of several hundred milliseconds, [26], [27], and there is a risk that a chunk can no longer be retrieved from DM because it has been forgotten, requiring WM-like access to multiple chunks causes interference.

At the start of every trial, participants were shown the subtraction condition (easy or hard), and were given a choice between two secondary tasks: tone-counting or tracking. In the tracking task [28] participants had to keep a moving dot within a circle using a trackball peripheral device. From a cognitive standpoint, the tracking task used both visual and manual resources, but WM is unlikely to be involved. In the tone-counting task tones were presented to participants through a pair of headphones. After completing the last digit of the subtraction task, participants were prompted to type in the number of tones they had heard. The tone-counting task required aural (auditory attention and processing) and WM resources, but not the visual resource.

There is no contention for WM resources when either tone-counting or tracking is combined with the easy subtraction task since the easy subtraction task does not require WM. However, since both tracking and subtraction require the visual and manual resources, threaded cognition predicts that easy subtraction is more compatible with tone-counting than with tracking. However, during a hard subtraction problem, there is significant overlap with each of the secondary tasks: The overlap with tracking is in the visual and manual resources, while the overlap with tone-counting is in the WM resource. Overlap in the WM resource is typically thought to be more disruptive than overlap in the visual and manual resources: visual and manual interference typically lead to delays in the order of 100–200ms, while reinstating WM contents from declarative memory consumes much more time and has a chance of failure [14], [26]. Based on these results, we predict that hard subtraction is more compatible with the tracking task than with the counting task. To summarize, the conditions form an interference gradient ranging from no interference (easy subtraction and tone-counting) to severe interference (hard subtraction and tone-counting), as a function of both resource overlap and resource type. We predict that if people adapt their choices to maximize utility during multitasking, they will choose task combinations that minimize interference.

We present three experiments aimed at investigating if and how behavior adapts to multitasking interference. In Experiment 1 we investigated whether task combinations led to the expected interference patterns and preference. Participants were first trained on all combinations of primary and secondary tasks, and were then given the choice of secondary tasks at the onset of every trial. In Experiment 2 we further investigate preference, as well as the learning behavior that leads to task preferences. We more strictly controlled the motivation to perform well, and participants were no longer trained on the task combinations before they were allowed to choose the second task freely. In Experiment 3 we further explore the degree of interference required for correct determination of ideal task combinations, by making the difference in interference between combinations more distinct.

## Experiment 1

### Participants

A total of 23 participants (16 female, *M_age_* = 21.0, age range: 18–24) were recruited for the experiment. This study was approved by the Ethical Committee Psychology of the University of Groningen, and written informed consent was obtained for all participants. Participants received €10 per hour for their participation. All participants had normal or corrected to normal vision.

### Materials and Methods

The setup consisted of the subtraction task with either tracking or tone counting. Participants were instructed to perform both tasks concurrently as well as they could. The subtraction was either an easy or hard problem. In all of the subtraction problems only the column to be solved was visible to the participants, with all other columns masked by hash marks. This was to prevent participants from using visual cues instead of WM to keep track of carries in the carry subtraction condition (cf. [29]). The left hand was positioned on the numeric keypad of a keyboard to solve the subtraction. Feedback on subtraction was only given during practice trials by coloring the number either green for correct or red for incorrect.

For tracking, the right hand was placed on the trackball. Feedback was audio-visual: each time the dot went outside the circle, the participant would be signaled by a beep, and the color of the circle would change from black to red. Given that motor skills can vary widely, we adapted task difficulty to the performance profiles of each participant: when the dot stayed in the circle for three seconds its movement speed would increase by 7% of the base speed. Whenever the dot went outside the circle, the movement speed would decrease by 14% of the base speed. Typically, this caused the dot to be inside the circle around 90% of the time.

In the tone-counting task participants had to click a trackball button with their right thumb when they heard a tone. This was done to keep both hands occupied, keeping participants from counting using their fingers. Participants had to respond at the end of the trial. If they reported the wrong number of tones when prompted, a buzzer sounded and the text ‘*Wrong*’ with the correct number of tones was displayed for six seconds. When answered correctly, the text ‘*Correct*’ would appear for half a second.

The study consisted of a single-task practice block (Block 0: 16 subtraction, divided into eight easy and eight hard trials, one tone counting, and one tracking trial) followed by a dual-task practice block (Block 1: 16 trials, with every concurrent task combination appearing four times). The order of combinations in Block 1 was counterbalanced between participants. This dual-task block with fixed combinations was added to give participants some experience with all possible combinations before introducing free choice of the secondary task. In the 48 trials of Block 2 the subtraction task alternated between easy and hard every two trials: before each trial the participants were shown the subtraction difficulty of the upcoming trial and could choose whether they wanted to perform tone counting or tracking concurrently with subtraction.

### Results

All reported *F*- and *p*-values are from repeated-measure ANOVAs, and all accuracy data were transformed with a logit transformation before performing ANOVAs. For the accuracy and latency data only Block 1 data were considered, as the number of trials per condition in Block 2 were unbalanced as the participants chose the task combinations. Table 1 summarizes the results.

**Table 1:** Summary of ANOVA results for Experiment 1.

Subtraction Task						
	Accuracy			Response Times		
Source	*F(3,66)*	*p*	*η_p_^2^*	*F(3,66)*	*p*	*η_p_^2^*
Type	89.35	<.001	.58	148.39	<.001	.83
Secondary Task	6.91	.011	.09	1.83	.181	.03
Type x Secondary Task	4.77	.033	.07	<1	-	-

Secondary Task						
	Tone-counting			Tracking		
Source	*F(1,22)*	*p*	*η_p_^2^*	*F(1,22)*	*p*	*η_p_^2^*
Type	29.76	<.001	.57	4.07	.056	.16

Type  =  Subtraction type.

**Figure 1:**
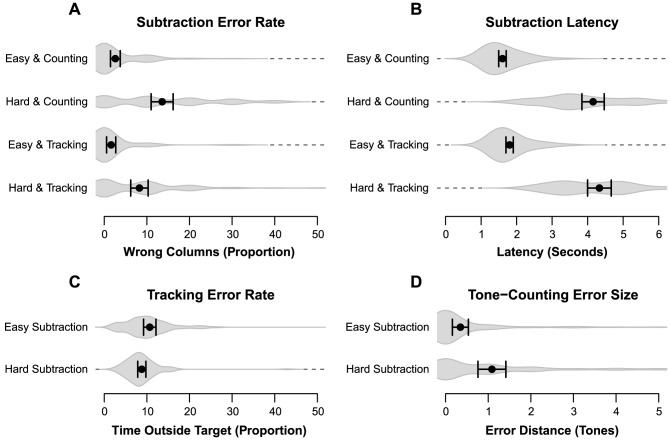
Performance results of Block 1 for Experiment 1. Averages for each condition are shown as a black dot, with a corresponding 95% confidence interval. The gray volume behind the averages is an estimate of the density, computed from the distribution of the data underlying the averages [29]. Panel A: Percentage of incorrect columns in a subtraction problem. Panel B: Latency on solving a single subtraction column. Panel C: Percentage of time outside the circle during the tracking trials. Panel D: Error distance of tone counting answers.

**Interference effects.** Analysis of the subtraction accuracy data (Figure 1A) shows a main effect of subtraction type (*F*(3, 66)  = 89.35, *p* <.001, 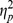 = 0.58), indicating that the accuracy of hard subtraction was lower than easy subtraction. There was an effect of secondary task (*F*(3, 66)  = 6.91, *p* = .011, 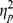 = 0.09), as the reduction in subtraction accuracy between easy and hard was smaller when subtraction was combined with tracking instead of tone-counting (6% vs. 10% accuracy reduction). There was also an interaction between type and secondary task (F(3, 66)  = 4.77, *p* = .033, 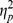 = 0.07): while easy subtraction performance is very similar for either secondary task, hard subtraction performance degrades more when combined with tone-counting.

A *post-hoc* Tukey honest significant difference (HSD) test showed that, apart from easy subtraction with tracking versus easy subtraction with tone counting, conditions were significantly different from each other (at the *p* <.001 level, except for hard subtraction with tracking versus hard subtraction with counting, which was significant at the *p* <.01 level). Contrary to tone-counting, and in line with the hypotheses, the addition of tracking did not affect subtraction accuracy much: participants performed only slightly worse compared to subtractions with no secondary task (a 0% error increase for easy subtractions, and a 1% error increase for hard subtractions). This indicates the visual interference resulted in only a mild performance reduction. For latency (Figure 1B) there is a main effect of subtraction type for solving a single column (*F*(3, 66) = 148.39, *p* <.001, 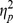 = 0.83), as hard subtractions take considerable more time to complete.

Examination of the performance on the tone counting and tracking tasks shows that while tracking accuracy (Figure 1C) is hardly affected by subtraction type (*F*(1, 22)  = 4.07, *p* = .056), tone counting (Figure 1D) is associated with lower accuracies when the subtraction task is hard: the mismatch between the given and the correct answer is larger (*F*(1, 22)  = 16.20, *p* <.001, 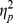 = 0.42). The effect of resource overlap is clear in the performance of the secondary task: tracking performance does not change much depending on the subtraction type, but participants make significantly more mistakes in tone-counting.

The tracking task adapts to the participant, so it is possible that a reduction in tracking speeds during hard subtraction trials causes the performance to remain steady. However, no difference was found between the average adapted tracking speeds of easy and hard trials as the average tracking speed during hard subtractions was 0.2% higher compared to easy subtractions (*F* < 1). As such, it is unlikely that the stable performance results from variances in tracking difficulty. This stability is expected if the interference between tracking and subtraction does not change when carries are introduced.

**Figure 2:**
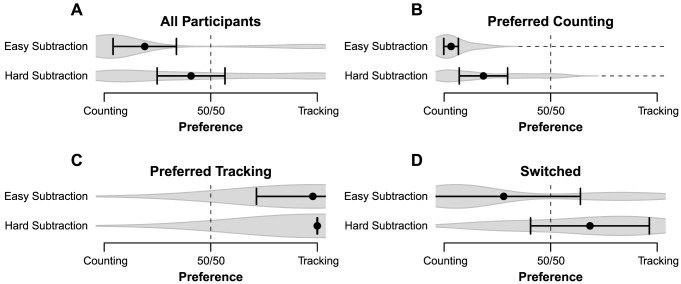
Secondary task choices for Experiment 1. Averages and 95% CI are plotted and the gray volume behind the averages is a plot of the estimated density of the underlying data [29] Panel A: Average choices for the best task combinations over all participants. Panel B, C and D: average combination choices for the participants that favored counting, tracking, and switching secondary task, respectively.

**Task preference.** The performance results are highly compatible with our initial prediction, and suggest that our paradigm is suitable to investigate choice adaptation to multitasking interference. An analysis of the Block 2 choice data (Figure 2A) shows that on average participants selected tone counting in 82% of the trials where the subtraction task had no carries. When faced with hard subtraction condition, there was a significant (*F*(1, 22)  = 8.13, *p* < 0.01, 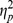 = 0.27) shift towards selecting the tracking task, which was chosen in 41% of all trials. This implies that participants had a strong preference for the optimal combination when subtraction was easy, but showed much less certainty when subtraction was hard, as secondary task preference was close to chance when subtraction was hard.

How well choices conform to the expected optimal task combinations can be described in terms of signal detection theory [30]. If we reason from the perspective of the tone-counting task, each secondary task choice can fall into one of four categories: hit (easy & tone-counting), false-alarm (hard & tone-counting), miss (easy & tracking), and correct rejection (hard & tracking). To measure detection performance, the true positive rate (TPR, also known as *sensitivity*) can be compared against the false positive rate (FPR, or *one minus specificity*). Within our paradigm, the TPR can be defined as the proportion of easy & tone-counting choices. Similarly, the FPR is the proportion of hard & tone-counting choices.

**Figure 3:**
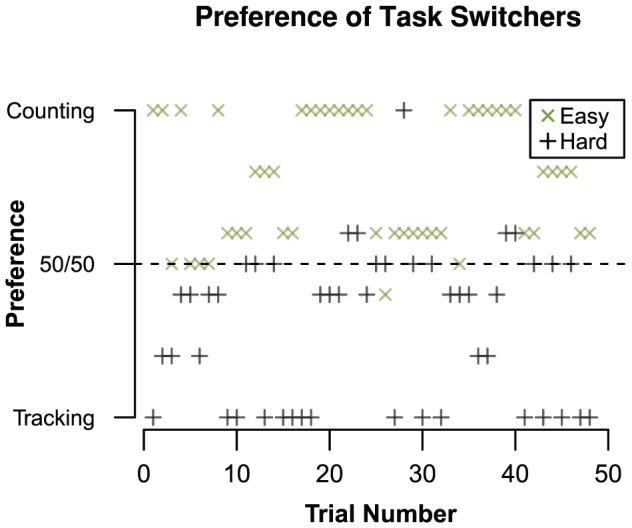
Change in preference over time for Experiment 1. Task preference over time is shown for the participants that switched between secondary tasks. Theoretically, the lowest interference is obtained when easy subtraction problems (“+”) are combined with counting, and hard subtractions (“x”) are combined with tracking.

We performed a two-dimensional hierarchical clustering of the participants based on their TPR and FPR scores [31], and found three distinct groups (shown in Figure 2B, 2C and 2D): participants who chose tone-counting almost exclusively (*n* = 14, or 61%), participants who chose tracking almost exclusively (*n* = 2, or 9%), and participants who, as predicted for sufficient interference sensitivity, switched secondary task (*n* = 7, or 30%). Choices of the participants who switched are in the direction of the expected combinations, but did not seem to show a learning effect: preferences over trials as presented in Figure 3 show no convergence toward the predicted optimal combinations during Block 2, as during later trials participants still combined hard problems with counting and easy problems with tracking.

## Discussion

With Experiment 1 our aim was to determine whether the predicted interference between certain combinations of tasks held, and whether or not people base their choice of tasks only on the tasks themselves, or also on the multitasking context. The behavioral results were in line with our predictions: the task combination with the highest expected interference, hard subtraction with tone-counting, leads to the lowest performance. This is followed by hard subtraction with tracking; the remaining two combinations result in the least interference, and thus in highest performance. This is consistent with the concept of overlap in resources leading to interference, and that WM interference is more detrimental to performance than visual interference. Of the secondary tasks, only tone-counting showed large interference effects; indicating that tracking and subtraction are quite compatible within this paradigm.

As the Block 1 performance results were within expectations, we predict that, if people adapted to multitasking interference, tone-counting would be chosen for easy subtractions, while tracking would be chosen for hard subtractions. The data showed a strong preference for tone-counting when subtraction was easy, but a much weaker preference for tracking when subtraction was hard: participants seem to adapt only partially. A closer look revealed large individual differences in decision behavior. Most participants always selected the same secondary task, even though it resulted in suboptimal performance. This indicates that for these participants the interference did not affect task utility enough to switch tasks strategically. The remaining participants showed strong adaptation to the different subtraction conditions by switching between secondary tasks. However, only a portion of that group made decisions that conformed to the optimal combinations. The other switching participants showed the general expected pattern, but did not converge to the optimal choices over time. It is possible these participants were still experimenting with task combinations to find the optimal solution when the experiment ended.

The results argue that most people do not adapt their choices to minimize the multitasking interference, at least not in the time available in the experiment. A possible explanation is that their preference for one of the tasks outweighed the possible advantage of avoiding multitasking interference. An additional potential benefit of always selecting the same secondary task is that it keeps things simpler, and offers more opportunity for speed improvements due to practice.

## Experiment 2

The goal of experiment 2 is to diminish the impact of preference for one of the two secondary tasks, thereby boosting the possible effect of multitasking context. Moreover, it will investigate whether learning occurred in the choice process.

Importantly, the experiment was set up to discourage participants from picking the same secondary task all the time. This was accomplished by changing the difficulty of the secondary tasks depending on the choices of the participant. After each trial, the difficulty of the chosen secondary task increased, while the difficulty of the not-chosen secondary task decreased. In order not to give participants any additional clues regarding the nature of the experiment, they were informed that the task difficulty could change, but not when and how this change would occur. We assume that increased difficulty of a secondary task decreases the motivation of participants to choose it, and we therefore expect to see more participants exhibit switching behavior. As such, it will not be the switching per se that will measure whether participants adapted their choices to the interference, but how much higher the proportion of optimal combinations (in line with the expected preferences) is compared to the proportion of switches that can be considered random (performed in order to keep difficulty manageable). As preferences were mostly stable after Block 1 of Experiment 1, we did not include a block with predetermined task combinations: this should result in a clear measurement of a possible learning effect for optimal task combinations. Furthermore, we increased the number of trials during which participants could choose a secondary task, in case convergence to optimal preferences takes longer than we previously anticipated. As shown by the previous experiment, free choice in task combinations can result in an unequal number of observations per condition, which leads to an unbalanced design. A solution would have been to stop the experiment only after each condition has been seen an equal number of times. However, in practice this could prove infeasible: as some participants never explore all possible combinations, the experiment might not end. Therefore the number of trials was kept at a fixed number.

### Participants

A total of 41 new participants (28 female, *M_age_* = 22.1, age range: 18–25) were recruited for the experiment. This study was approved by the Ethical Committee Psychology of the University of Groningen, and written informed consent was obtained for all participants. Participants received €10 per hour for their participation. All participants had normal or corrected to normal vision.

### Materials and Methods

All three tasks used in the setup were identical to those in the previous experiment. Participants performed the same practice block used in Experiment 1. After the practice trials the participants were presented with a single block of 72 trials in which the subtraction condition was randomized such that in every four trials the participants would see both subtraction types twice. The difficulty of the secondary task depended on the history of choices: if the secondary task chosen in trial *n* is the same as that of trial *n*-1, the difficulty of that task will be increased after the trial. The difficulty of both secondary tasks had limits: from the starting difficulties *DC_s_* and *DT_s_* (reflecting the difficulty of the “C”ounting and the “T”racking task respectively) both tasks could increase and decrease by a maximum of 7 steps, resulting in a total of 15 difficulties per task. For tracking, one step was a change of 10% of *DT_s_*, (taken as the average adapted tracking speed in Experiment 1, minus one standard deviation) leading to a speed range from 0.3*DT_s_* to 1.7*DT_s_*. In similar fashion, one difficulty step for tone-counting was a change of 0.2 seconds in average tone interval, giving an average tone interval range of 0.5 to 3.5 seconds. Note that the tracking adaptation using in Experiment 1 was not used in Experiment 2.

### Results

As the number of observations per condition was inherently unbalanced due to the design of the paradigm, we used linear mixed-effects models instead of ANOVAs to interpret the results. Table 2 summarizes the results.

**Table 2:** Summary of mixed-effects model results for Experiment 2.

Subtraction Task						
	Accuracy			Response Times		
Source	*β*	*z*	*p*	*β*	*t*	*p*
Type	–2.30	–23.81	<.001	2.09	36.92	<.001
Secondary Task	–1.05	–6.07	<.001	–0.03	1.58	.113
Type x Secondary Task	0.83	7.05	<.001	–0.04	<1	-
Difficulty	–0.53	–4.88	<.001	0.27	3.82	<.001

Secondary Task						
	Counting Accuracy			Tracking Accuracy		
Source	*β*	*z*	*p*	*β*	*z*	*p*
Type	–1.10	–2.76	.005	0.18	2.14	.032
Difficulty	1.03	6.67	<.001	–47.09	–26.27	<.001
Type x Difficulty	–0.15	<1	-	–4.28	–2.33	.020

Type  =  Subtraction type, difficulty  =  Secondary task difficulty.

**Figure 4:**
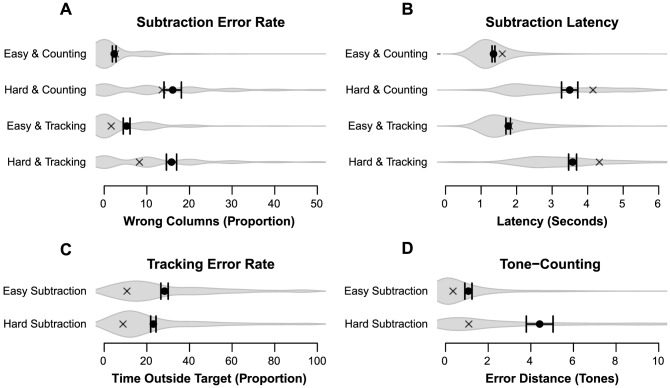
Performance results for Experiment 2. Similarly to Figure 1, averages and 95% CI are plotted and the gray volume behind the averages is a plot of the estimated density of the data [29]. Observed averages of Experiment 1 are plotted as gray crosses. Panel A: Percentage of incorrect columns in a subtraction problem. Panel B: Latency on solving a single subtraction column. Panel C: Percentage of time outside the circle during the tracking trials. Panel D: Error distance of tone counting answers.

The performance within each group bear a strong qualitative resemblance to the results of Experiment 1 (represented by the gray crosses in each panel of Figure 4 for easy comparison), indicating that the task combinations that minimize interference were not influenced by changes to the paradigm. Subtraction accuracy (Figure 4A) shows a main effect for subtraction type (*β* = –2.30, *z* = –23.81, *p*<.001), which argues that easy subtractions were indeed easier than hard subtractions. Furthermore, there was an interaction between subtraction type and secondary task (*β = *0.27, *z* = 2.84, *p*<.001), indicating that subtraction accuracy decreased less from easy to hard when tracking was used as secondary task. In Figure 4B, subtraction latency shows a pattern very similar to accuracy. Latency only shows a main effect of subtraction type (*β* = 2.09, *t* = 36.92, *p*<.001), just as we observed in Experiment 1: hard subtractions took much longer to complete than easy subtractions.

Tracking performance (Figure 4C) is numerically higher in the hard condition (*β* = 0.18, *z* = 2.14, *p* = .032). There was a large effect of tracking difficulty on accuracy (*β* = –47.09, *z* = –26.27, *p*<.001), as well as an interaction between tracking difficulty and subtraction type (*β* = –4.28, *z* = –2.33, *p*<.020), indicating that increased difficulty led to lower tracking performance when subtraction was hard. This is likely due to the higher latency of hard subtractions, which left less time for tracking. Much like tracking accuracy, the size of tone-counting errors (Figure 4D) depended strongly on tone-counting difficulty (*β* = –2.35, *t* = –3.71, *p*<.001). While there was no main effect of subtraction type on the size of the errors (*β* = 0.33, *t*<1), this main effect was found for the number of correct counts (*β* = –1.10, *z* = –2.76, *p* = .005). This indicates that there were more errors during hard subtractions, but the spread of the error distance remained the same between the two subtraction types. Overall, the qualitative performance differences are very similar to what was found in Experiment 1, with changes in task difficulty having a significant negative impact on each task.

**Figure 5:**
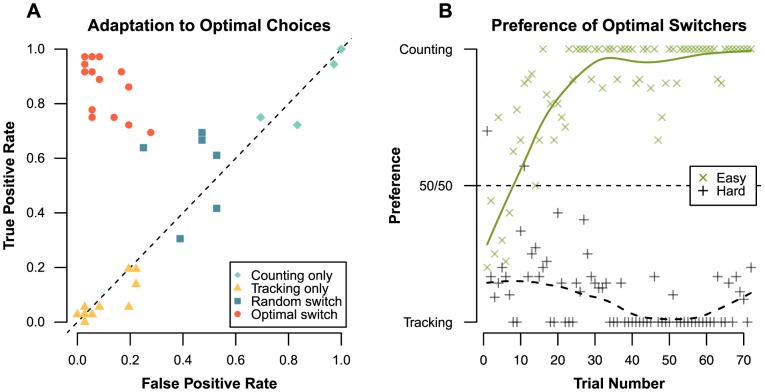
Adaptation to interference for Experiment 2. Panel A: Sensitivity of each participant, grouped according to ta hierarchical clustering of the preference data. Panel B: Change in preference over trials of the optimally switching participant group.

Similar to the analysis of Experiment 1, we used a hierarchical clustering [31] on the TPR and FPR scores to group participants. Two distinct groups were found: participant who switched regularly between secondary tasks (*n* = 21, or 51%, number of switches: *M_switch_* = 31.57, *SD_switch_* = 11.57), and participants who hardly switched at all (*n* = 20, or 49%, number of switches: *M_switch_* = 4.05, *SD_switch_* = 4.49). By plotting the TPR against the FPR we visualize samples in Receiver Operating Characteristic (ROC) space [30] as presented in Figure 5A: points closer to the top-left corner indicate a greater adaptation to the optimal combinations, points on the diagonal mean chance-level task choices, and points closer to the bottom-right equal lower-than-chance adaptation. Against expectation, the majority of the not-switching group (*n* = 15, or 75% of the not-switchers) only used the tracking task (bottom left corner), which is an inversion of the tone-counting preference (top right corner) found in Experiment 1. Of the participants that switched, those clustered around the middle of the diagonal showed a random switching pattern (*n* = 6, or 40% of the switchers). The remaining switchers are in the top left corner, and conformed to the expected optimal choices (*n* = 9, or 60% of the switchers). This means that of the participants who switched, the majority adapted their choices to the multitasking interference, and that out of all the participants slightly over a fifth adapted their behavior (9 out of 41, or 22%).

For the optimally switching participants, the preference over time is presented in Figure 5B. Optimal switchers did not immediately exhibit the predicted switching behavior: the preferences are unstable during early trials. About halfway through the experiment the secondary task preferences converged to the predicted minimal interference combinations, giving strong evidence that adapting behavior to multitasking interference progresses as a learning curve. Preferences for both difficulties start out with a bias for tracking as secondary task. This seems in line with the not-switching participants, who also largely prefer tracking. However, this is a departure from the counting bias found in Experiment 1, and could be caused by the absence of the fixed combinations block, which could influence preference before the start of the free-choice block.

### Discussion

Based on the hypothesized explanation that the penalty for suboptimal performance was too low in Experiment 1, we predicted that a larger proportion of participants would show switching behavior in Experiment 2. The increased proportion of switching participants supports this hypothesis. Furthermore, due to the removal of the block with fixed task combinations, we obtained a better description of changes in preference over time.

Although some participants chose the same secondary task exclusively, it is clear that when participants are made more aware of the increased costs of suboptimal combination of tasks, their concurrent multitasking decisions improve. A possible explanation why some participants never switched is the prioritization of the subtractions: the second task becomes less relevant, and performance on that task is largely ignored in favor of high performance on the subtraction, even though overall performance suffers. Prioritizing the concurrently performed task that is perceived as most important has been observed in other research as well [32]. Furthermore, participants could have been prioritizing a single secondary task: instead of having to learn two additional tasks, they focused on improving their performance in either tone-counting or tracking, ignoring the other task.

The majority of participants that focused on one secondary task chose tracking. This is not a bad choice: tracking is optimal with hard subtraction, and only slightly worse than tone-counting for easy subtractions. The small difference between tracking and tone-counting for easy subtractions might have caused a substantial number of participants to be stuck in this sub-optimal solution. Of the switching participants, about half eventually converge toward task preferences that minimize interference. However, this learning curve takes considerable time, at approximately the same rate for both subtraction conditions.

The data of Experiment 2 imply that a fifth (9 out of 41) of participants adapt their choices to minimize interference, and almost half of all participants performed sub-optimally. Several factors could have contributed to this result: for easy subtractions the difference in performance between tone-counting and tracking might have been too small to be noticed by the majority of participants. Furthermore, the random-switching and not-switching behavior might also have arisen due to a lack of instruction regarding mechanism that determined secondary task difficulty. In Experiment 3 we investigate how these factors affect adaptation to multitasking interference.

## Experiment 3

**Figure 6:**
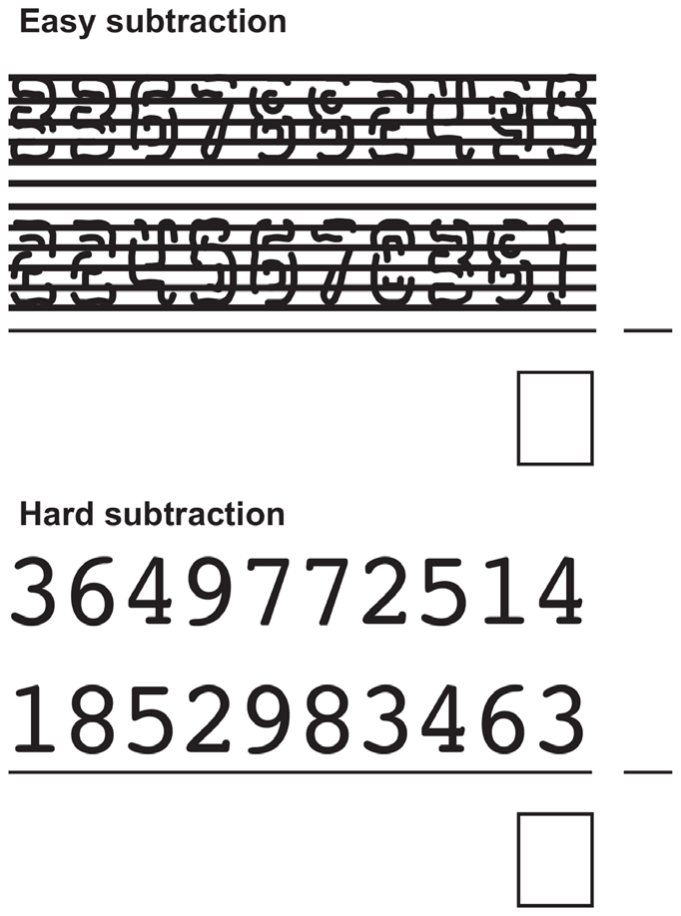
Subtraction task used in Experiment 3. The easy subtraction was visually degraded to increase the cost of visual attention switching when combined with tracking. In the actual experiment all columns except the one to be solved were masked with # marks.

The effects of instruction clarity and interference strength were investigated by introducing two changes to the paradigm. First, before the experiment the participants were informed how the secondary task difficulty changed depending on their choices. Second, to increase the visual interference between tracking and easy subtraction, the easy subtraction task was visually degraded (see Figure 6), thereby increasing the visual processing load. This change should make the interference difference for secondary tasks more explicit in the easy condition, while maintaining the difference between secondary tasks in hard subtractions. We predict that these changes will increase the proportion of participants that switch secondary task, compared to Experiment 2.

### Participants

For the final experiment 28 new participants (13 female, *M_age_* = 20.9, age range 17-25) were recruited. This study was approved by the Ethical Committee Psychology of the University of Groningen, and written informed consent was obtained for all participants. Participants received €10 per hour for their assistance, and all participants had normal or corrected to normal vision.

### Materials and Methods

Apart from the changes listed here, the paradigm was similar to Experiment 2. The largest change was the visual degradation: the font was changed from the highly legible ‘Courier’ to the more difficult to process ‘Mlurmly’, and lines with a thickness of three pixels were drawn across the numbers at regular intervals (Figure 6). By using the legibility measure proposed by Van Rossum [33], we determined that the new easy subtraction text was 53% less legible than the old text: while still readable, processing the new easy subtraction task should take slightly longer now. In addition, before the start of the experiment participants were instructed that changes in difficulty were based on their history of task choices. Finally, the main trial block was slightly shorter, as it now consisted of 60 trials.

### Results

As in Experiment 2, the performance data was analyzed using a mixed-effects model to account for the differences in the number of observations per condition. Table 3 summarizes the results.

**Table 3:** Summary of mixed-effects model results of Experiment 3.

Subtraction Task						
	Accuracy			Response Times		
Source	*β*	*z*	*p*	*β*	*t*	*p*
Type	–0.07	<1	-	2.17	28.06	<.001
Secondary Task	–0.27	–2.13	.033	1.21	8.56	<.001
Type x Secondary Task	0.98	9.73	<.001	–0.53	–4.88	<.001
Difficulty	0.17	1.52	.129	–0.06	<1	-

Secondary Task						
	Counting Error Size			Tracking Accuracy		
Source	*β*	*t*	*p*	*β*	*z*	*p*
Type	3.55	4.66	<.001	0.66	10.98	<.001
Difficulty	–0.93	–3.24	.001	–77.59	–46.14	<.001
Type x Difficulty	–0.90	–2.23	.026	–4.49	–2.91	.004

Type  =  Subtraction type, difficulty  =  Secondary task difficulty.

**Figure 7:**
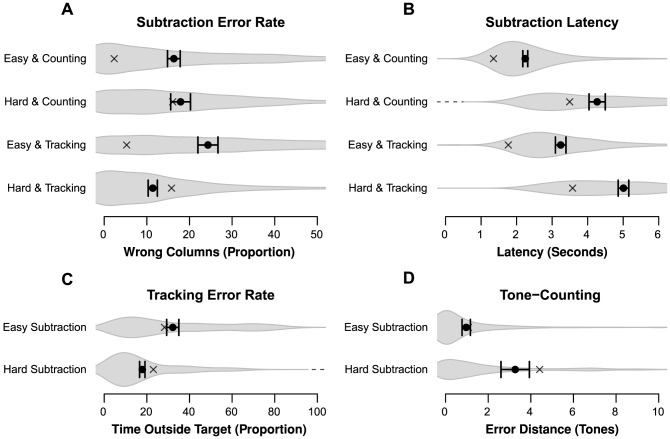
Performance data for Experiment 3. Averages and 95% CI are plotted and the gray volume behind the averages is a plot of the estimated density of the underlying data [29]. Observed averages of Experiment 2 are plotted as gray crosses. Panel A: Percentage of incorrect columns in a subtraction problem. Panel B: Latency on solving a single subtraction column. Panel C: Percentage of time outside the circle during the tracking trials. Panel D: Error distance of tone counting answers.

When comparing the results of Experiment 3 (error bars in Figure 7) with the results of Experiment 2 (see Figure 5 for the full data, or the crosses in Figure 7 for easy comparison), a number of notable differences can be observed: Participants were slower at performing the subtraction task, and easy subtraction performance was worse due to the visual degradation we introduced. There was an interaction between subtraction type and secondary task (*β* = –0.27, *z* = –2.13, *p* = .033), as tone-counting caused a larger accuracy loss when subtraction changes from easy to hard, whilst subtraction combined with tracking shows the opposite pattern. Thus, the visual degradation change seems to have had the desired effect on the subtraction task. Furthermore, subtraction difficulty seems to have become more similar between both types compared to the earlier experiments. For latency, we see a main effect for subtraction type (*β* = 2.17, *t* = 28.06, *p*<.001): hard subtractions required more time than easy subtractions. There is a main effect for secondary task (*β* = 1.21, *t* = 8.56, *p*<.001) as well, as tracking leads to longer subtraction latency than tone-counting does. However, the interaction between subtraction type and secondary task (*β* = –0.53, *t* = 4.88, *p*<.001) suggests that for tracking the latency increased less when the subtraction becomes hard when compared to tone-counting.

Tracking shows a clear main effect of subtraction type (*β* = 0.60, *t* = 4.79, *p*<0.001), as well as a main effect of secondary task difficulty (*β* = –95.15, *t* = –23.36, *p*<0.001): Compared to Experiment 2 difficulty was better controlled on average as instructions regarding the changing secondary task difficulty were more clear, but the visual degradation in the easy subtractions reduced time available for the tracking task. Overall tone-counting performance has improved a bit compared to Experiment 2, and clearly shows main effects for subtraction type (*β* = 3.55, *t* = 4.66, *p*<0.001).

**Figure 8:**
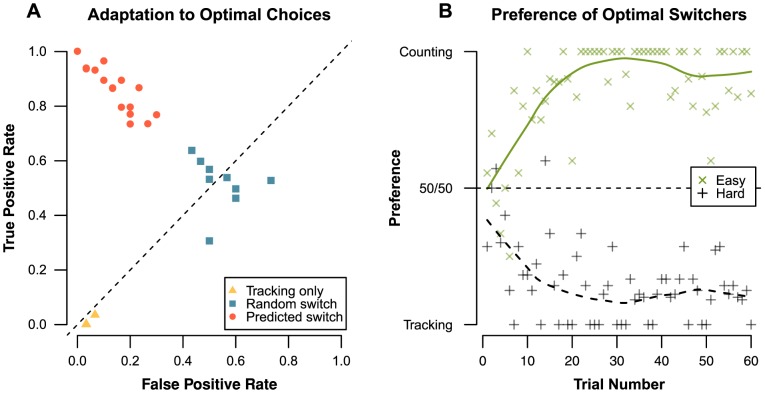
Adaptation to interference for Experiment 3. Panel A: Detection sensitivity for each participant group, as determined by the hierarchical clustering. Panel B: Change in preference over time for the participants that show the predicted preferences.

As in the first two experiments, a hierarchical clustering was performed to identify distinct groups of behavior (Figure 8A). As only a few participants kept selecting tracking almost all the time (*n* = 3, or 11%), the changes to the paradigm seem to have had the anticipated effect by preventing the majority of participants from choosing suboptimal task combinations. This means that in accordance with our prediction, most participants displayed switching behavior (*n* = 25, or 89%). Almost two-thirds of the switching participants are located in the top left corner, indicating that they showed the predicted choice preferences (*n* = 16, or 64% of the switchers), while the rest switched more or less randomly by choosing the optimal preferences at chance level (*n* = 9, or 36% of the switchers). Hence, the proportion of expected to random switching has changed only slightly compared to Experiment 2. Within the predicted switching group, the convergence to the expected preference (Figure 8B) seems to occur at the same rate as in Experiment 2. Thus, the larger interference difference between secondary tasks in the easy subtraction condition does not seem to have had any effect on the learning speed.

### Discussion

In Experiment 3 almost all participants show switching behavior, which is a clear difference from Experiment 2. However, both the ratio of optimal to random switching (60% vs. 64% of switching participants) as well as the convergence to the predicted preferences was comparable to the results of Experiment 2. This would imply that while the changes in the experiment promoted switching between secondary tasks, they did not affect how secondary task utility was influenced by the paradigm: the severity of interference has only a small effect on adaptation of choices to the interference. Given that the proportion of participants switching randomly to participants switching in an optimal manner is very similar in two experiments, this seems to be a robust finding. This could mean that there are people who are less sensitive to multitasking interference, or that some were simply not motivated enough to take the effort to find optimal combinations. However, if motivation were truly an issue, then it would be less effortful to simply pick the same task every time instead of reasoning about which task to pick at each trial, so it seems more likely that the differences in interference were too subtle for the randomly switching participants.

The rate of adaptation towards optimal combinations also seems to suggest that utility dynamics were not affected by the changes in the paradigm. While discovering optimal combinations took time, the speed of learning was very similar to Experiment 2. Thus, an increased difference in interference severity between two selectable dual-task alternatives did not affect the rate at which task utility changed. This could imply that the change in interference was too subtle to cause any changes in adaptation rate, or that the task utility for non-optimal tasks was higher at the start of the experiment for this batch of participants. Alternatively, it could be that some sort of evidence accumulation process, which is independent of utility, influences how fast preferences converge to optimal combinations.

In conclusion, as Experiment 2 and 3 are in agreement regarding how many people adapt to multitasking interference and how fast this adaptation occurs, we have strong evidence that a small majority of people will adapt their choice behavior to reduce interference between tasks, and that this adaptation has a considerable learning curve.

## General Discussion

We investigated whether people adapt their decisions to minimize the interference found in a concurrent dual-task. We found that most people are indeed sensitive to subtle effects of interference. A series of three experiments show increasing levels of choice adaptation when preference for a single secondary task is ruled out as much as possible. However, it takes time before the choices are fully adapted to the multitasking context: combinations that minimize interference are not recognized immediately.

Despite our attempts to discourage a preference for a single secondary task, some participants did not exhibit any switching behavior. It seems that the preference for a certain task is so strong that our manipulations are not yet sufficient to entice them to explore other possibilities. To put this in terms of utility, there are two possibilities why some participants do not switch: it could be that the a priori utility of a one secondary task is so high that it cannot be surpassed by another secondary task in the time it took the experiments to complete. The second possibility is that for some participants the utility for secondary tasks is simply not affected by the manipulations of the paradigm. This could be the case if secondary task utility was ignored in order to concentrate on the primary subtraction task. If subtractions were prioritized in such a way, it could be that the change in utility was ignored, or utility was no longer affected in a meaningful way by the effects of task interference. In our experiment we did not investigate the effect of task prioritization on choice adaptation, so this area is left open for further exploration.

Of all the participants that switched secondary task, some did so randomly. The simplest explanation is that while these participants were not able to detect what combinations have the lowest interference, they did try to adapt to the increasing difficulty of trials. This explanation would mean their task choices were not based on the utility of any of the involved tasks. A more intricate version of this account that does take task utility in account is that it could be another possible response to prioritizing the subtraction task: by keeping the secondary task difficulty low its impact on the subtraction task remains small.

Prioritizing the primary subtraction task seems to be a recurring explanation for not adhering to the optimal switching behavior. Even though participants were instructed to perform both tasks equally well, the constant presence of subtractions might have created a subconscious bias toward that task. Unfortunately, the current work offers no way to infer the subtraction priority for individual participants. Establishing the effect of priority on secondary task preference would be a valuable addition that supplements the current work, and therefore and interesting topic for further investigation.

Alongside task priority, the learning rate of optimal combinations also leads to new questions. Surprisingly, increasing the difference in inference between competing combinations did not show an effect on learning rate. From a utility standpoint this is suboptimal: A greater difference in interference should result in a greater difference in reward for either choice, with the reward of the better choice being higher. This should lead to the utility of the corresponding task to increase more rapidly, and as such the preference for that task should increase faster as well. As of yet, the cause of the static learning rate is still an open issue.

Finally, it is important to highlight one difference with everyday multitasking. While participants had some freedom in task choices, the choice of whether or not to multitask was fixed: participants could not choose to perform just one task. As such, our findings are only relevant to situations where multitasking is strongly promoted or required. More generalizable conclusions about adaptation to interference would require a paradigm where one of the options available to the participants is to focus solely on a single task.

In conclusion, it seems that people are in principle able to make correct judgments about the costs of multitasking, although it might take some time and experience. Thus, the adage stating that people are poor at multitasking might need to be amended.

### Authors’ Notes

Data collected from the experiments can be obtained from the corresponding author.
